# Supplementary material for: Identification of Two Independent COL5A1 Variants in Dogs with Ehlers–Danlos Syndrome
Source: Genes (Basel). 2019 Sep 21;10(10):731. doi: 10.3390/genes10100731 (PMC6826881; doi:10.3390/genes10100731)
Supplement: Supplementary file 1 [file genes-10-00731-s001.pdf]

Table S1. *COL5A1*:c.4711G>A genotypes of 493 dogs from 66 different dog breeds.

Breed Abbreviation	Breed	Number of dogs	Genotype		
			G/G	A/G	A/A
**AI**	**Akita Inu**	8	8	0	0
AC	Australian Cattle Dog	8	8	0	0
AD	Alpine Dachsbracke	8	8	0	0
AP	Appenzeller Mountain Dog	8	8	0	0
AK	Australian Kelpie	7	7	0	0
AU	Australian Shepherd	8	8	0	0
BA	Barbet	8	8	0	0
BO	Barsoi	7	7	0	0
BG	Bavarian Mountain Hound	6	6	0	0
BE	Beagle	8	8	0	0
BD	Bearded Collie	8	8	0	0
BS	Bernese Mountain Dog	7	7	0	0
BC	Border Collie	8	8	0	0
BN	Boston Terrier	7	7	0	0
CE	Cairn Terrier	8	8	0	0
CS	Cão da Serra de Aires	8	8	0	0
CC	Chinese Crested Dog	8	8	0	0
CW	Chow Chow	7	7	0	0
CR	Curly Coated Retriever	7	7	0	0
TW	Czechoslovakian Wolfdog	8	8	0	0
DH	Dachshund	8	8	0	0
DW	Deutscher Wachtelhund	7	7	0	0
EL	Elo	8	8	0	0
EN	Entlebucher Mountain Dog	8	8	0	0
EU	Eurasian Dog	8	8	0	0
FR	Flat Coated Retriever	6	6	0	0
FB	French Bulldog	8	8	0	0
DS	German Shepherd	6	6	0	0
KS	German Spitz (Kleinspitz)	5	5	0	0
MS	German Spitz (Mittelspitz)	7	7	0	0
GW	German Wirehaired Pointer	7	7	0	0
RS	Giant Schnauzer	8	8	0	0
GR	Golden Retriever	6	6	0	0
DD	Great Dane	8	8	0	0
GY	Greyhound	8	8	0	0
GD	Groenendael	4	4	0	0
JT	Hunting Terrier	6	6	0	0
IT	Irish Terrier	8	8	0	0
JR	Jack Russell Terrier	8	8	0	0
WS	Keeshound (Wolfsspitz)	7	7	0	0
KF	Kromfohrländer	8	8	0	0
LA	Labrador Retriever	8	8	0	0
LR	Lagotto romagnolo	8	8	0	0
LN	Landseer	8	8	0	0
LB	Leonberger	8	8	0	0
MA	Malinois	8	8	0	0
MB	Miniature Bullterrier	6	6	0	0
RP	Miniature Pinscher	8	8	0	0
NF	Newfoundland	8	8	0	0
NW	Norwich Terrier	8	8	0	0

NR	Nova Scotia Duck Tolling Retriever	8	8	0	0
PE	Perro de Agua Espanõl	8	8	0	0
PO	Polski Owczarek Nizinny	7	7	0	0
ZS	Pomeranian (Zwergspitz)	7	7	0	0
GP	Poodle (Grosspudel)	8	8	0	0
RR	Rhodesian Ridgeback	8	8	0	0
SB	Saint Bernard	8	8	0	0
NS	Schapendoes	8	8	0	0
SC	Scottish Terrier	7	7	0	0
SY	Siberian Husky	7	7	0	0
SS	Shetland Sheepdog	8	8	0	0
SG	Sloughi	8	8	0	0
TT	Tibetan Terrier	7	7	0	0
WW	West Highland White Terrier	8	8	0	0
WH	Whippet	7	7	0	0
BB	White Swiss Shepherd	8	8	0	0
	**Total**	**493**	**493**	**0**	**0**
